# Immunohistochemical Assessment of Phosphorylated mTORC1-Pathway Proteins in Human Brain Tumors

**DOI:** 10.1371/journal.pone.0127123

**Published:** 2015-05-19

**Authors:** Patrick N. Harter, Lukas Jennewein, Peter Baumgarten, Elena Ilina, Michael C. Burger, Anna-Luisa Thiepold, Julia Tichy, Martin Zörnig, Christian Senft, Joachim P. Steinbach, Michel Mittelbronn, Michael W. Ronellenfitsch

**Affiliations:** 1 Edinger Institute, Institute of Neurology, University of Frankfurt am Main, Frankfurt am Main, Germany; 2 German Cancer Consortium (DKTK), Heidelberg, Germany; 3 German Cancer Research Center (DKFZ), Heidelberg, Germany; 4 Department of Neurosurgery, University of Frankfurt am Main, Frankfurt am Main, Germany; 5 Senckenberg Institute of Neurooncology, University of Frankfurt am Main, Frankfurt am Main, Germany; 6 Georg-Speyer-Haus, Institute for Tumor Biology and Experimental Therapy, Frankfurt am Main, Germany; University Hospital of Navarra, SPAIN

## Abstract

**Background:**

Current pathological diagnostics include the analysis of (epi-)genetic alterations as well as oncogenic pathways. Deregulated mammalian target of rapamycin complex 1 (mTORC1) signaling has been implicated in a variety of cancers including malignant gliomas and is considered a promising target in cancer treatment. Monitoring of mTORC1 activity before and during inhibitor therapy is essential. The aim of our study is to provide a recommendation and report on pitfalls in the use of phospho-specific antibodies against mTORC1-targets phospho-RPS6 (Ser235/236; Ser240/244) and phospho-4EBP1 (Thr37/46) in formalin fixed, paraffin embedded material.

**Methods and Findings:**

Primary, established cell lines and brain tumor tissue from routine diagnostics were assessed by immunocyto-, immunohistochemistry, immunofluorescent stainings and immunoblotting. For validation of results, immunoblotting experiments were performed. mTORC-pathway activation was pharmacologically inhibited by torin2 and rapamycin. Torin2 treatment led to a strong reduction of signal intensity and frequency of all tested antibodies. In contrast phospho-4EBP1 did not show considerable reduction in staining intensity after rapamycin treatment, while immunocytochemistry with both phospho-RPS6-specific antibodies showed a reduced signal compared to controls. Staining intensity of both phospho-RPS6-specific antibodies did not show considerable decrease in stability in a timeline from 0–230 minutes without tissue fixation, however we observed a strong decrease of staining intensity in phospho-4EBP1 after 30 minutes. Detection of phospho-signals was strongly dependent on tissue size and fixation gradient. mTORC1-signaling was significantly induced in glioblastomas although not restricted to cancer cells but also detectable in non-neoplastic cells.

**Conclusion:**

Here we provide a recommendation for phospho-specific immunohistochemistry for patient-orientated therapy decisions and monitoring treatment response.

## Introduction

**Fig 1:**
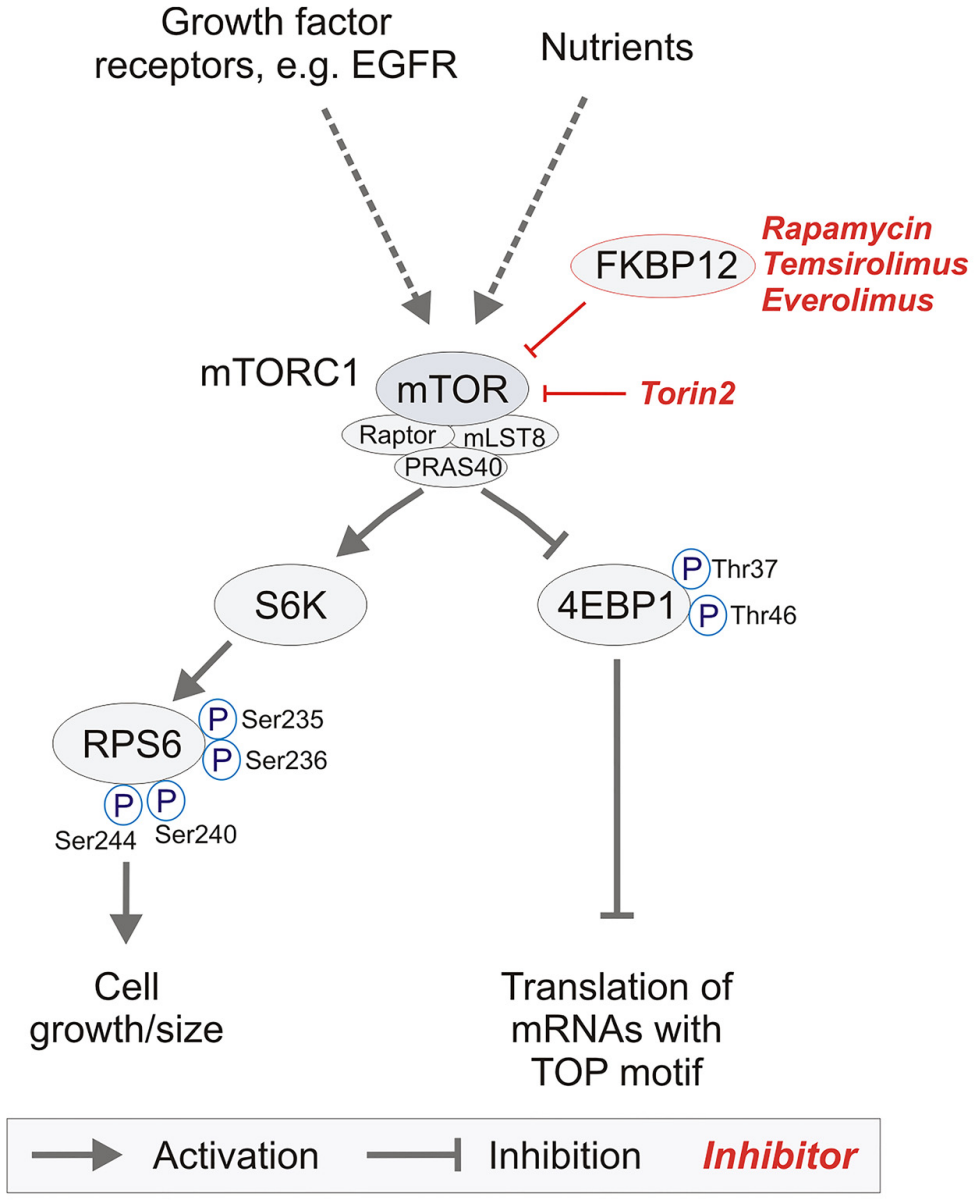
Overview of essential intracellular members of the mTORC1-pathway and respective targeted therapies. Signaling from growth factor receptors such as EGFR or a nutrient rich environment leads to an activation of the mTOR-pathway, while nutrient depletion inhibits signaling. The mTORC1-complex consists of the mTOR-protein itself and the subunits raptor (regulatory associated protein of mTOR), mLST8 (mammalian lethal with sec thirteen 8) and PRAS40 (proline-rich Akt1 substrate 1). Activation of the pathway leads to phosphorylation of the ribosomal protein S6 at phospho-sites Ser235/236 and Ser240/244 by the S6-kinase (S6K). Additionally, the translational repressor 4EBP1 (eukaryotic initiation factor 4E binding protein-1) is phosporylated at Thr37/Thr46, thereby relieving its translational inhibition and promoting translation especially of mRNAs with a TOP motif [14]. First generation mTORC1-inhibitors are rapamycin and its derivatives temsirolimus and everolimus. Mechanistically these compounds bind the intracellular adaptor protein FKBP12 to form an allosteric mTORC1 inhibitory complex. 4EBP1 phosphorylation though mTORC1-dependent is largely resistant to the mTORC1 inhibitor rapamycin and its derivatives. Recently, ATP-competitive mTORC (2^nd^ generation) inhibitors like torin1 and torin2 which are efficient in dephosphorylating 4EBP1 have been developed [15].

While classical chemotherapeutic drugs are still the major backbone in cancer treatment, more and more targeted therapies enter clinical application. The concept of targeted therapy aims at interfering with individual key oncogenic drivers that can be exploited as specific drug targets. In cancer, few major oncogenic signaling pathways have been identified. One of these pathways is regulated by the mammalian target of rapamycin complex 1 (mTORC1). mTORC1 functions as a protein kinase that controls protein biosynthesis thereby contributing to cell growth. Its activity is regulated by various stimuli (Fig 1)—activators include nutrient redundancy as well as signaling from growth factor receptors which causes an Akt-mediated relief of hamartin/tuberin (TSC1/2)-mediated mTORC1 inhibition. TSC1/2 are known tumor suppressors, loss of function mutations in these genes cause tuberous sclerosis complex (TSC), a fairly frequent tumor syndrome that includes the development of subependymal giant cell astrocytomas (SEGAs) [1].

Classical allosteric inhibitors of mTORC1 signaling derive from rapamycin, a drug isolated from Streptomyces hygroscopicus with fungicidal activity [2], which has later been shown to act as an immunosuppressive and antitumor drug [3,4]. Clinical development focuses on the rapamycin derivatives everolimus (RAD-001) and temsirolimus (CCI-779) with improved pharmacokinetics that have been approved for some cancer entities including SEGAs (in the U.S.A), renal cell carcinoma and mantle cell lymphoma [5–8]. Allosteric mTORC1 inhibitors rely on the intracellular adaptor protein FKBP12 for their action (Fig 1). So called 2nd generation mTORC1-inhibitors are characterized by their ability to interfere with the ATP-binding motif of the mTOR-protein in a competitive manner to inhibit signaling from both mTOR complexes (mTORC1&2) as described for the potent mTORC-inhibitor torin2 [9]. While mTORC1 signaling is considered an important oncogenic pathway in gliomas, first clinical trials in glioblastoma (GBM) patients with rapamycin or its derivatives as monotherapy were rather sobering [10–12]. Several reasons have been discussed and include insufficient target inhibition or primary resistance by activation of alternative signaling pathways. These challenges are met by the design and clinical testing of 2nd generation mTORC1 inhibitors and the quest for patient subgroups that benefit from mTORC1 inhibitor treatment indicating a potential need for predictive biomarkers and/or appropriate combinatorial treatments. Therefore, accurate monitoring of mTORC1 activity before and during mTORC1 inhibitor therapy is instrumental to pave the way of these drugs into the clinic. Currently, the large number of ongoing trials targeting the mTOR-pathway with mono- or combinatorial therapies in brain tumor patients highlights the biological relevance of mTOR and the hope that lies on its pathway’s inhibition (Table 1 provides an overview of selected clinical trials in brain tumor patients). Most of these clinical trials, however, do not explicitly point to the inclusion of biomarkers, and neglect a pre-screening to measure the extent of mTORC1 activation within the tumors of each individual tumor patient. Nevertheless, there is rising evidence that the mTOR-signaling pathway especially in case of recurrent GBM plays an important role in tumor relapse, as a recent study demonstrated a high rate of mutations of members of the mTOR-pathway after temozolomide treatment [13]. Therefore, not just in these patients in vivo testing of mTOR-signaling activity is strongly needed to generate a rationale for mTOR-targeted therapy.

**Table 1:** Overview of a selection of clinical trials targeting the mTOR-pathway in brain tumor patients.

*Entity*	*Compound*	*Inclusion*	*Phase*	*NCT number*	*Status*
low grade glioma	RAD-001	chemotherapy refractory low grade glioma; children/adults	II	NCT01158651	Active, not recruiting
low grade glioma	RAD-001	recurrent low-grade glioma; adults	II	NCT00823459	Recruiting
low grade glioma	RAD-001	recurrent low-grade glioma; children/adults	II	NCT01734512	**Recruiting** ^**1**^
low-grade glioma	RAD-001	chemotherapy refractory low grade glioma; children/adults	II	NCT00782626	Completed *no results available*
high-grade glioma	CCI-779+ Perifosine	recurrent/progressive high-greade glioma; adults	I/II	NCT01051557	Active, not recruiting
low-grade glioma	RAD-001 +/- Temozolomide	low-grade glioma; adults	II	NCT02023905	Recruiting
high-grade glioma	RAD-001 +/- Sorafenib	recurrent high-grade glioma; adults	I/II	NCT01434602	Recruiting
high-grade glioma	Rapamycin +Erlotinib	recurrent high-grade glioma; adults	I/II	NCT00509431	Completed *no results available*
low-grade glioma	RAD-001	recurrent or progressive low grade glioma; adults	II	NCT00831324	**Recruiting** ^**2**^
high-grade glioma	CCI-779+Erlotinib	resurrent high-grade glioms; adults	I/II	NCT00112736	Completed no results available
low-grade glioma	Rapamycin +Tarceva	low-grade glioma with or without NF1-type children/adults	I	NCT00901849	Enrolling by invitation
high-grade glioma	CCI-779	high-grade glioma; adults	I/II	NCT00022724	Completed no results available
Glioblastoma /Gliosarcoms	Rapamycin +Erlotinib	recurrent Glioblastoma / Gliosarcoma; adults	II	NCT00672243	Completed has results
Glioblastoma /Gliosarcoms	RAD-001 +Gleevec +Hydroxyurea	recurrent Glioblastoma / Gliosarcoma; adults	I	NCT00613132	Completed no results available
high-grade glioma	**AZD8055**	recurrent high grade glioma; adults	I	NCT01316809	Completed no results available
Ependymoma	RAD-001	recurrent or progressive ependymoma; children, adults	II	NCT02155920	**Not yet recruiting no results available** ^**3**^
Glioblastoma	RAD-001	recurrent Glioblastoma; adults	II	NCT00515086	**Terminated has results** ^**4**^
SEGA	RAD-001	SEGAs with Tuberous Sclerosis Complex; children/adults	I/II	NCT00411619	Active, not recruiting
SEGA	RAD-001	SEGAs with Tuberous Sclerosis Complex; children/adults	III	NCT00789828	Active, not recruiting has results
Gliolastoma Prostate Cancer	RAD-001 +Gefitinib	progressive Glioblastoma; adults	I/II	NCT00085566	**Completed no results available** ^**5**^
Glioblastoma	RAD-001 +Temozolomide	newly diagnosed Glioblastoma; adults	I/II	NCT00553150	**Active, not recruiting** ^**6**^
Meningioma	RAD-001 +Bevacizumab	refractory, progressive intracranial meningioma; adults	II	NCT00972335	**Active, not recruiting** ^**7**^
Meningioma /Schwannoma	RAD-001	NF-type 2 / vestibular schwannoma / meningioma; adults	0	NCT01880749	Recruiting
NF-type 2	RAD-001	NF-type 2 patients; children and adults	II	NCT01419639	Active, not recruiting

Some study protocols point on the issue of using tissue biomarkers for additional evaluation of treatment-response as follows:

^1^Exploration of associations with pS6 positivity and outcome is planned

^2^Correlation of phosphorylated PKB/Akt and PTEN expression with response is planned

^3^Correlation of tumor objective response rate to established immunohistochemical biomarkers of mTOR pathway activation, including pS6, p4EBP1, pPRAS40, pp70S6K and PTEN is planned

^4^Use of Biomarkers: Phosphatase and tensin homolog (PTEN) and Epidermal Growth Factor Receptor (EGFR)

^5^ Comparison of clinical outcome compared to immunohistochemical markers related to the EGFR and PTEN-PI3K-AKT pathways at baseline is planned

^6^ Evaluation of laboratory variables (phospho-Akt, PTEN status, MGMT expression and promoter methylation status)

^7^Correlation of the activity of the treatment regimen with expression of selected intra-tumoral biomarkers

(AZD8055: ATP-competitive mammalian target of rapamycin kinase inhibitor)

The two best characterized phosphorylation targets of mTORC1 are 4EBP1 and S6 kinase 1 (S6K1). S6K1 phosphorylates ribosomal protein S6 (RPS6) which is an essential component of the 40S ribosomal subunit. RPS6 phosphorylation influences cell growth and progresses in an ordered sequential fashion starting with Ser236 followed by Ser235, Ser240, and Ser244 [16,17]. While Ser240/244 phosphorylation is undetectable in S6 kinase knockout mice, Ser235/236 phosphorylation still occurs, most likely mediated by p90RSK in a mitogen-activated protein kinase (MAPK)-dependent fashion [18]. The molecular consequences of RPS6 phosphorylation are still the subject of ongoing research, so far no data about differential effects with regard to the phosphorylation sites of RPS6 (Ser235/236 vs. Ser 240/244) are available. 4EBP1 is a translational regulator and functions by sequestering eukaryotic translation initiation factor 4E (eIF4E) from the translation initiation machinery. Phosphorylation of 4EBP1 also occurs in an ordered fashion [19]. Thr70 is phosphorylated first, followed by Thr37/46 and by Ser65. While Thr70 phosphorylation has no effect on 4EBP1 binding of eIF4E, it is the Thr37/46 phosphorylation that relieves its inhibition *in vivo* [19]. mTORC1-mediated Thr37/46 phosphorylation of 4EBP1 seems to be largely resistant to allosteric mTORC1 inhibition by rapamycin and its derivatives temsirolimus and everolimus.

In summary, phospho-4EBP1 as well as phospho-RPS6 are potentially suitable biomarkers for mTORC1-signaling in routine diagnostics.

Studies trying to identify patient subgroups which might profit from a targeted therapy by phospho-specific IHC have already been performed in different tumor subtypes [11,12,20–22], however, up to now none of these markers entered routine histo- or neuropathological diagnostics. Especially in case of GBM this might be related to the negative results of some clinical studies, which did not confirm a prognostic or even predictive role of the tested phospho-signals [23]. Furthermore, the detection of phosphoproteins in FFPE tissue is strongly dependent on the grade of dephosphorylation, as demonstrated for phospho-AKT, therefore reliable detection of phospho-signals in routinely collected specimens constitutes a great challenge for (neuro-)pathologists [24].

We hypothesize that mTORC1-signaling is detectable and upregulated in FFPE tissue specimens deriving from malignant brain tumors, while its detection might strongly depend on tissue processing including fixation time and tissue size. Additionally, we hypothesize that mTORC1-signaling is induced during tumor progression in glioma patients. Furthermore, as mTORC1-complex activation can be demonstrated by distinct phosphorylation sites of 4EBP1 and RPS6, we were asking which phosphorylation sites might be most reliable as biomarkers for mTORC1-activation for (I) pre-treatment patient stratification or (II) therapy monitoring.

In summary, we here present pitfalls and provide a recommendation for immunohistochemical assessment of mTORC1 signaling using phospho-specific antibodies against phospho-RPS6 (Ser235/236), phospho-RPS6 (Ser240/244), phospho-4EBP1 (Thr37/46) in routine FFPE brain tumor specimens.

## Materials and Methods

### Tissue specimen and tissue processing

We analyzed FFPE tissue from archived brain tumors derived from the UCT tumor bank (Goethe-University, Frankfurt am Main, Germany, member of the German Cancer Consortium (DKTK), Heidelberg, Germany and German Cancer Research Center (DKFZ), Heidelberg, Germany). The use of patient material for this study was endorsed by the local ethics committee (Ethics committee UCT Frankfurt / Goethe University Frankfurt am Main, Germany: project numbers: GS 4/09; SNO_01–12, GS 4/09; SNO-06-2014). All specimens were fixed in 4% paraformaldehyde (formalin) including 3 samples of subependymal giant cell astocytomas. Besides whole mount sections of malignant brain tumors such as glioblastomas and brain metastases, We analyzed primary and secondary brain tumors spotted as tissue micro arrays (TMAs) into paraffin blocks, including brain metastases of: melanoma (n = 3), non-small cell lung cancer (NSCLC n = 5), breast carcinoma (n = 2), small cell lung cancer (SCLC n = 2), Colorectal carcinoma (n = 1). Primary malignant brain tumors: Anaplastic astrocytoma (n = 2), anaplastic oligodendroglioma (n = 3), medulloblastoma (n = 2), glioblastoma (n = 11). For statistical analyses we analysed FFPE TMAs of brain tumor patients (n = 15) which had a relapse during their clinical course and underwent tumor progression (Table 2). Tissue included the following specimens: normal appearing grey matter (NAGM) tissue of surgical specimens (n = 7), diffuse astrocytomas (n = 4), anaplastic astrocytoma (n = 7), anaplastic oligo-astrocytoma (n = 4), glioblastoma (GBM) (n = 15).

**Table 2:** Overview of brain tumor patients for statistical analysis.

*ID*	*primary tumor*	*recurrent tumor*
1	diffuse astrocytoma WHO grade II	GBM WHO grade IV
2	anaplastic astrocytoma WHO grade III	GBM WHO grade IV
3	diffuse astrocytoma WHO grade II	GBM WHO grade IV
4	anaplastic astrocytoma WHO grade III	GBM WHO grade IV
5	diffuse astrocytoma WHO grade II	GBM WHO grade IV
6	anaplastic astrocytoma WHO grade III	GBM WHO grade IV
7	anaplastic astrocytoma WHO grade III	GBM WHO grade IV
8	anaplastic astrocytoma WHO grade III	GBM WHO grade IV
9	anaplastic oligo-astrocytoma WHO grade III	GBM WHO grade IV
10	diffuse astrocytoma WHO grade II	GBM WHO grade IV
11	anaplastic oligo-astrocytoma WHO grade III	GBM WHO grade IV
12	anaplastic astrocytoma WHO grade III	GBM WHO grade IV
13	anaplastic oligo-astrocytoma WHO grade III	GBM WHO grade IV
14	anaplastic oligo-astrocytoma WHO grade III	GBM WHO grade IV
15	anaplastic astrocytoma WHO grade III	GBM WHO grade IV

For evaluation of dephosphorylation grade, we fixed GBM tissue in a dephosphorylation timeline of 35, 40, 50, 60, 80, 100, 130 and 230 minutes after resection. These samples did not exceed a size larger than 1.0 x 0.6 x 0.6 cm. Evaluation of early dephosphorylation events was also performed using the freshly harvested glioma cell line LNT-229 (see paragraph preparation of FFPE cell pellets). Embedding was performed using a standard automated embedding procedure.

### Statistical analysis

We analysed the amount of phospho-RPS6 (Ser235/236; Ser240/244)-, phospho-4EBP1 (Thr37/46)- as well as RPS6- and 4EBP1-positive cells as percentage of all cells. For statistical analyses we analyzed the groups of WHO grade II, III and IV gliomas and compared expression levels with each other and normal appearing grey matter specimens. We used non-parametric Kruskal-Wallis test with Dunn`s multiple comparisons post-test. A significance level was set for p<0.05. Statistical analysis was performed using GraphPad Prism 5 and 6 (GraphPad Software, La Jolla, CA, U.S.A.).

### Preparation of FFPE cell pellets

GBM LNT-229 cells [25] were cultured in DMEM containing 10% fetal calf serum (FCS) as well as 1% of penicillin and streptomycin or under serum-free (FCS-free) conditions. For evaluation of inhibition of the mTOR-pathway, we treated the cells with 100 nM rapamycin and 100 nM torin2 for 24 h in serum-free DMEM. For all experiments cell pellets were generated from subconfluent LNT-229 cells which were kept in T175 culture flasks. Cells were harvested with trypsin and centrifuged at 320 g to generate a cell pellet in 15 ml Falcon tubes which then was fixed for 24–72 h in 4% formalin.

For evaluation of early dephosphorylation events, we kept centrifuged cell pellets without culture medium in 15 ml Falcons and fixed the cells after 0, 15, 30, 60 minutes with 4% formalin. Embedding was performed using a standard automated embedding procedure.

### Primary culture of glioblastoma cells under sphere conditions

Primary culture was generated from a fresh human glioblastoma specimen and cultured in DMEM-F12 containing 20 ng/ml of each recombinant epidermal growth factor (EGF) and basic fibroblast growth factor 2 (bFGF2) (Reliatech, Wolfenbüttel, Germany). Additionally, we supplemented the culture medium with 20% BIT admixture 100 supplement (Pelo Biotech, Planegg/Martinsried, Germany). Cells were cultivated in flasks coated with 5 mg/ml laminin (Sigma, Deisenhofen, Germany) for 3 h. Cell pellets were generated using the above mentioned protocol except that accutase was used to harvest the cells.

### Immunocytochemistry / Immunofluorescence

Immunocyto- and immunohistochemistry for all antibodies was performed using freshly cut 3 μm thick slides from FFPE tissue or FFPE cell pellets on the automated IHC staining system Discovery XT (Roche/Ventana, Tucson, Arizona, USA). The following antibodies were used: phospho-RPS6 (Ser235/236) diluted 1:400 (D57.2.2.E; Cell Signaling, Boston, USA); phospho-RPS6 (Ser240/244) diluted 1:2000 (D68F8; Cell Signaling); phospho-4EBP1 (Thr37/46) diluted 1:1000 (236B4; Cell Signaling). For analysis of total protein counterparts we used an antibody detecting RPS6 diluted 1:100 (5G10; Cell Signaling) and an antibody detecting 4EBP1 diluted 1:400 (53H11; Cell Signaling). The staining procedure for phospho-specific antibodies on the Discovery XT contained heat treatment of the slides (75°, 95° and 100° Celsius), cell conditioning (CC1 for phospho-RPS6 (Ser235/236) and phospho-4EBP1 (Thr37/46); CC2 for phospho-RPS6 (Ser240/244)) and incubation of primary antibodies for 32 minutes. As secondary antibodies we used Universal secondary antibodies (Univ 2ndary AB) for 32 minutes (phospho-RPS6 (Ser240/244)) as well as OMap anti-Rb HRP (Multimer HRP) for 16 minutes (phospho-RPS6 (Ser235/236) and phospho-4EBP1 (Thr37/46)). As substrate we used diaminobenzidine (DAB) as DAB CM (phospho-RPS6 (Ser235/236) and phospho-4EBP1 (Thr37/46)) as well as DAB D (phospho-RPS6 (Ser240/244)) followed by a drop of H_2_O_2_. Copper was added for signal enhancement as Copper CM (phospho-RPS6 (Ser235/236) and phospho-4EBP1 (Thr37/46)) and Copper D (phospho-RPS6 (Ser240/244)) for 4 minutes. Slides were counterstained with hematoxylin and mounted.

For immunofluorescent stainings we used the following primary antibody concentrations on tissue micro arrays (see paragraph tissue specimen and tissue processing): phospho-RPS6 (Ser235/236) diluted 1:200 (D57.2.2.E; Cell Signaling); phospho-RPS6 (Ser240/244) diluted 1:1000 (D68F8; Cell Signaling); phospho-4EBP1 (Thr37/46) diluted 1:500 (236B4; Cell Signaling). Additionally, we used a mouse anti-GFAP antibody diluted 1:500 (G3893; Sigma Aldrich, Saint Louis, USA) as well as a mouse anti-CD68 antibody diluted 1:250 (M0876; Dako, Glostrup, Denmark). As secondary antibodies we used goat anti-mouse Alexa 568 or goat anti-rabbit Alexa 488 antibodies (Life technologies, Carlsbad, CA, USA) both diluted 1:500.

### Immunoblotting

Cells were washed and scraped with ice-cold PBS. Lysates were prepared using lysis buffer P containing protease and phosphatase inhibitors (#1861280; Thermo Scientific, Dreieich, Germany). 10 μg of protein per condition were subjected to SDS-PAGE analysis. Membranes were probed with antibodies against phospho-4EBP1 (Thr37/46) (236B4; Cell Signaling), phospho-RPS6 (Ser 240/244) (D68F8; Cell Signaling), phospho-RPS6 (Ser 235/236) (D57.2.2.E; Cell Signaling), RPS6 (5G10; Cell Signaling), 4EBP1 (53H11; Cell Signaling) or actin (# sc-1616 Santa Cruz Biotechnology, Dallas, Texas, USA). The secondary anti-rabbit and anti-goat antibodies were purchased from Santa Cruz Biotechnology (Dallas, Texas, USA). Chemiluminescence solution was used for detection and was set up with 1 ml solution A (200 ml 0.1 M Tris-HCl pH 8.6, 50 mg Luminol), 100 μl solution B (11 mg p-hydroxycurmarinacid, 10 ml DMSO) and 0.3 μl H_2_O_2_ (30%).

### Clinical trials

Literature research regarding ongoing clinical trials using rapamycin or derivates was performed accessing www.clinicaltrials.org (14^th^ august 2014). The following search terms were used: sirolimus AND glioma; everolimus AND glioma; temsirolimus AND glioma; tacrolimus AND glioma; sirolimus AND meningioma; everolimus AND meningioma; temsirolimus AND meningioma; tacrolimus AND meningioma.

## Results

### mTORC1-signaling can be assessed reliably by immunocytochemistry in glioma cell lines as well as in subependymal giant cell astrocytomas

**Fig 2:**
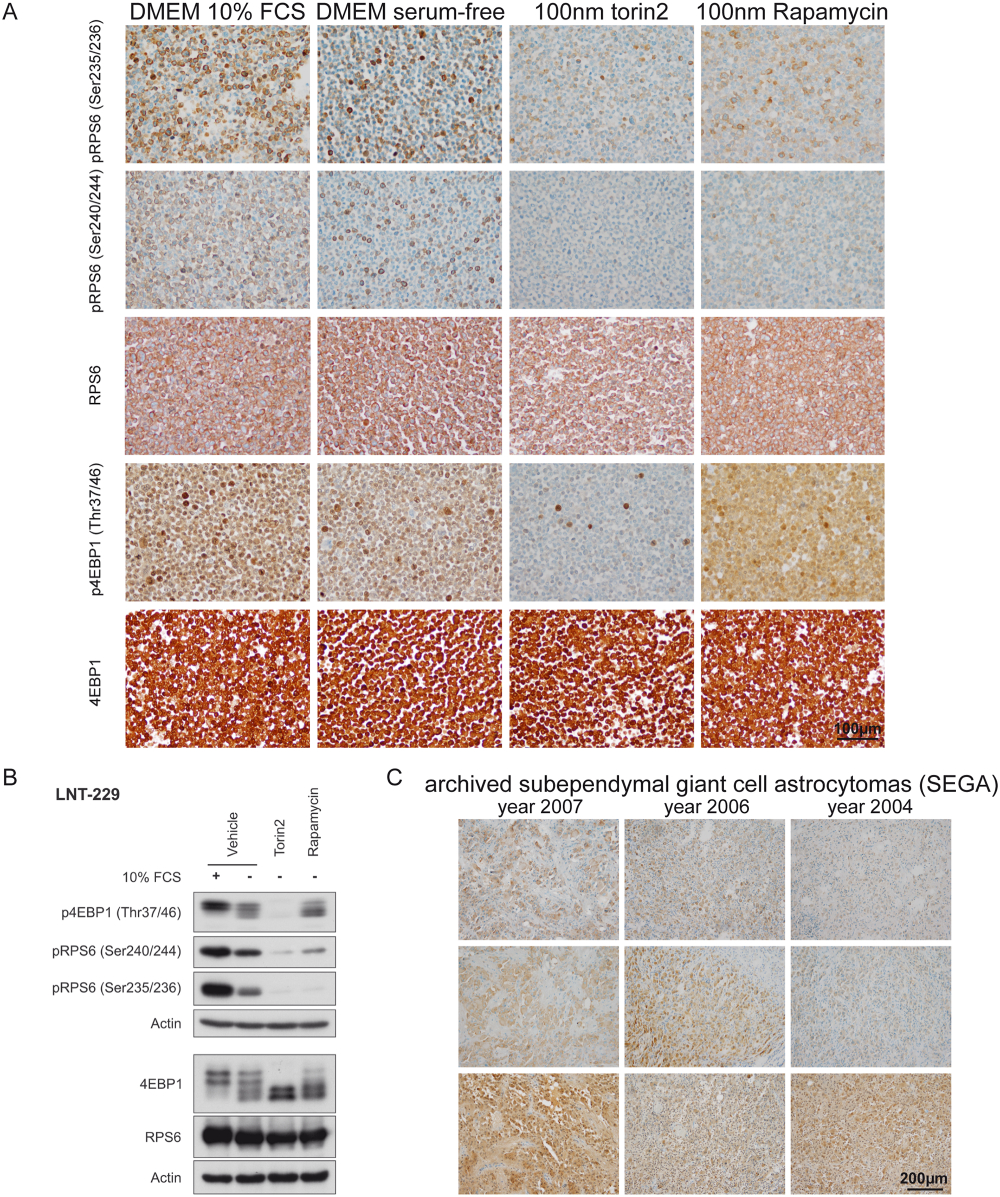
Establishment of mTORC1-specific immunohisto- and immunocytochemistry. (A) LNT-229 glioma cells cultured in DMEM containing 10% FCS and 25 mM glucose displayed strong signals in phospho-specific ICC stainings with all tested antibodies, while serum-free culture condititons resulted in reduced signal intensity and frequency. Treatment with the ATP-competitive mTORC-inhibitor torin2 resulted in a profound reduction of the phospho-spcific signal for all antibodies tested, while treatment with rapamycin only reduced the signal of RPS6 phospho-sites. Total RPS6 and 4EBP1 protein counterparts did not show considerable differences in the tested conditions (B) Immunoblotting using the same antibodies, cell lines and culture conditions confirmed the ICC results presented in (A), 4EBP1 exists in states of different electrophoretic mobility depending on its phosphorylation state [19]. (C) Subependymal giant cell astrocytomas (SEGA) served as a positive control for human routine diagnostic specimens. Although mTORC1-signaling was detectable in SEGA tumor cells, the staining intensity was considerably reduced in freshly cut archived FFPE material especially for the RPS6 phospho-sites.

We observed strong signals for phospho-RPS6 (Ser235/236), phospho-RPS6 (Ser240/244) and phospho-4EBP1 (Thr37/46) in the glioma cell line LNT-229 cultured in DMEM (glucose levels 25 mM) containing 10% serum (Fig 2A and 2B). While the staining for phospho-RPS6 (Ser235/236) and phospho-RPS6 (Ser240/244) was mostly restricted to the cytoplasm of cells we detected phospho-4EBP1 (Thr37/46) also in tumor cell nuclei. Withdrawal of serum led to a considerable reduction of signal intensity and frequency in cultured tumor cells. To prove that the detected signal was dependent on mTOR-signaling, we used cells exposed to the specific inhibitors rapamycin and torin2 as controls. While the treatment of LNT-229 cells with torin2 almost led to eradication of the ICC signal, we observed a reduction of specific staining in rapamycin-treated cells only with the antibodies against phospho-RPS6 (Ser235/236) and phospho-RPS6 (Ser240/244) (Fig 2A and 2B). In all tested conditions total protein counterparts did not change. In case of immunoblotting

4EBP1 phosphorylation states are associated with different states of electrophoretic mobility [19]. The biologically relevant Thr37/46 phosphorylation, however, is detectable in all mobility states. When cells are cultured in the presence of 10% FCS 4EBP1 is present in states of reduced electrophoretic mobility (slower migrating bands). Serum withdrawal increases the amount of higher mobility states (faster migrating bands), rapamycin in contrast to torin2 has only little effect in reducing 4EBP1 in its slower migrating states (Fig 2B). RPS6 protein amount is slightly increased when cells are cultured in medium containing 10% FCS. mTOR inhibitor treatment has no effect on the RPS6 protein amount (Fig 2B).

Subependymal giant cell astrocytomas (SEGAs) are known to depend on mTORC1-signaling, therefore archived tissue derived from SEGAs served as a positive control for the in vivo detection of phospho-specific IHC signal (Fig 2C). We detected a strong staining in tumor cells with all tested antibodies, while especially perivascular cells and most endothelial cells did not show any staining. No obvious staining gradient was detectable. To assess whether the age of the FFPE-tissue might be relevant for detection of mTORC1-induced phospho-signals we analyzed SEGAs resected in 2007, 2006 and 2004 (Fig 2C). The staining intensity with the antibodies against phospho-RPS6 (Ser235/236) and phospho-RPS6 (Ser240/244) was considerably reduced especially in case of the 10 year old block deriving from surgical material from 2004. Total protein level detection of 4EBP1 and RPS6 also showed a considerable reduction in case of the 10 year old block (S1 Fig).

### Time to formalin-fixation as potential pitfall in ICC and IHC detection of pRPS6 and p4EBP1

**Fig 3:**
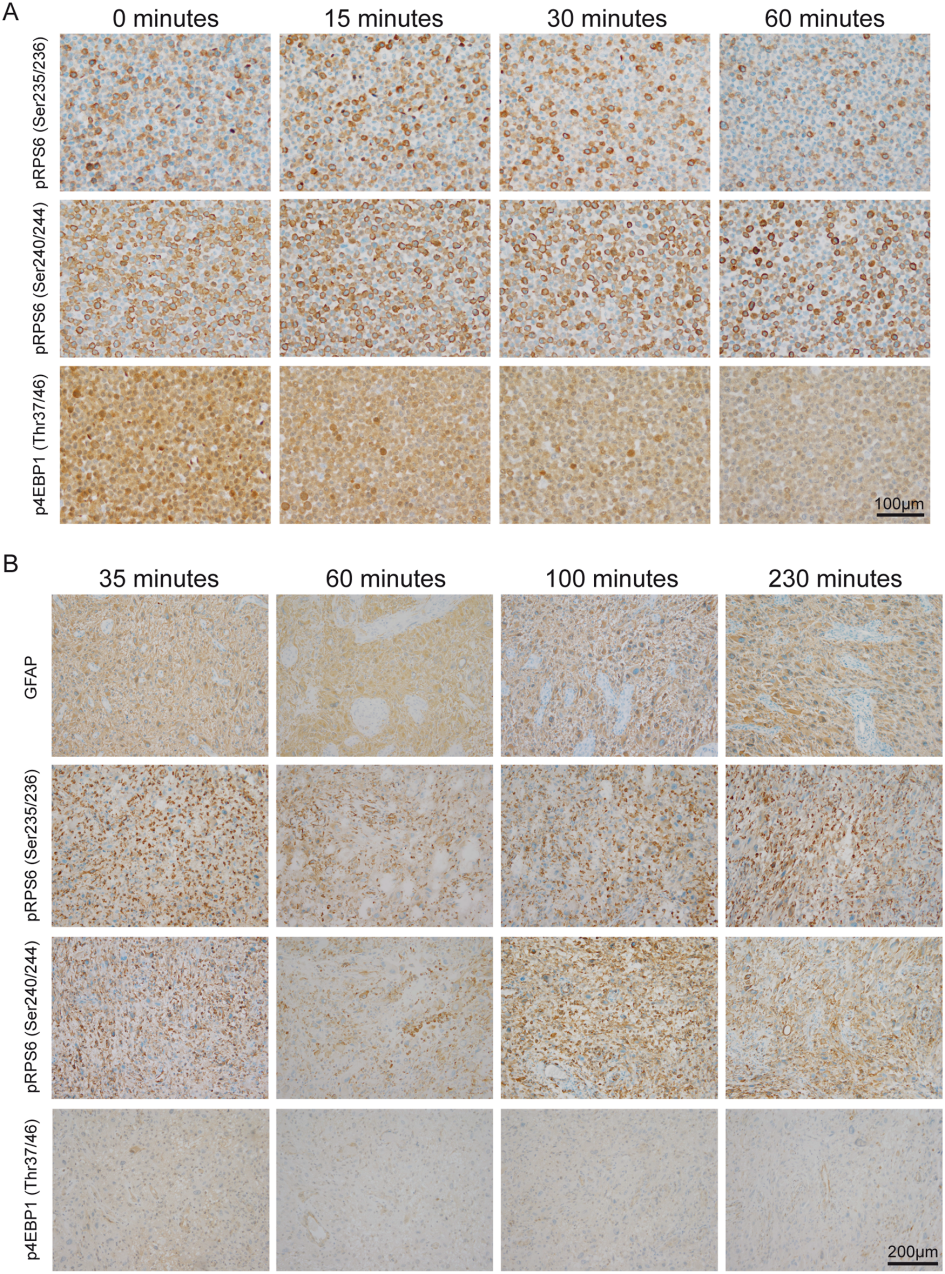
Timeline of in vivo and in vitro assessment of phospho-signals before formalin-fixation. (A) *In vitro* detection of phospho-RPS6 (Ser235/236), phospho-RPS6 (Ser240/244) and phospho-4EBP1 (Thr37/46) 0–60 minutes after harvesting LNT-229 glioma cells. Cells were kept at room temperature without medium in an open 15 ml plastic tube. Considerable reduction of p4EBP1 (Thr37/46) signal intensity was observed after 60 minutes. (B) *In vivo* detection of phospho-signals 35–230 minutes after surgical resection of a GBM. As a control to show general immunogenicity of the tissue samples we used an antibody against GFAP. While staining signals of phospho-RPS6 (Ser235/236) and phospho-RPS6 (Ser240/244) did not show considerable decrease over the respective time intervals, we hardly detected any signal for phospho-4EBP1 (Thr37/46) in the tissue specimens.

We investigated whether the time to formalin-fixation affected the signal intensity, both *in vitro* and *in vivo* (Fig 3A and 3B). Interestingly, phospho-RPS6 (Ser235/236) and phospho-RPS6 (Ser240/244) signal intensities hardly showed any reduction in LNT-229 cells within the first 60 minutes without fixation, whereas staining frequency was mildly affected. In contrast, the staining intensity of phospho-4EBP1 (Thr37/46) showed a considerable decrease within the first 60 minutes (Fig 3A). When analyzing total protein counterparts of RPS6 and 4EBP1 we did not observe considerable changes throughout the depicted timeline (S1 Fig). To assess whether time to fixation of *in vivo* tumor tissue alters mTORC1-phospho-signals, we incubated unfixed tumor material from a GBM at room temperature in time intervals between 35–230 minutes after resection (Fig 3B). Phospho-signals for both RPS6-antibodies did not show considerable changes in staining intensity or frequency over the whole time period. However, we observed only a very faint staining with the antibody against phospho-4EBP1 (Thr37/46) most likely due to physiological dephosphorylation within the first 35 minutes (Fig 3B), as total protein counterparts did not show considerable changes (S1 Fig).

### Phospho-specific stainings show a fixation-dependent gradient in vivo and in vitro

**Fig 4:**
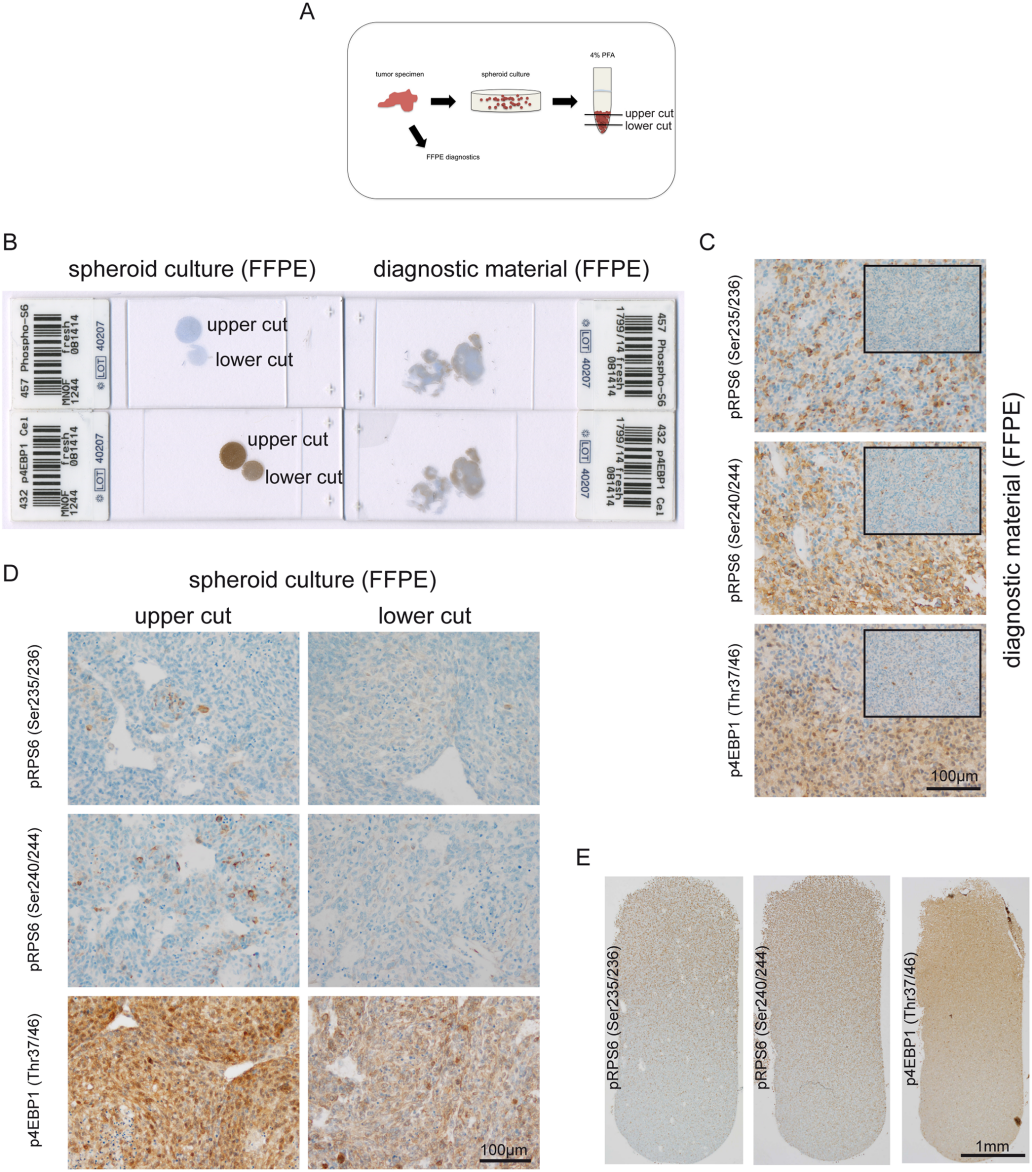
Decreasing in vivo and in vitro phospho-signals as a consequence of slow formalin diffusion. (A) Glioblastoma tissue was processed for routine diagnostics, and in addition, a primary spheroid culture from the same tissue was generated. The spheroid culture was spun down and supernatants were aspirated. We slowly added 4% formalin to the 15 ml tube to avoid a resuspension of cells. The fixated pellet was cut at the upper side as well as in the middle of the conus. Finally, the material was processed for paraffin-embedding according to the standard diagnostic procedure. (B) Macroscopic overview of 4 stained sections with cell pellet and corresponding glioma specimen. (C) Detection of phospho-RPS6 (Ser235/236), phospho-RPS6 (Ser240/244) and phospho-4EBP1 (Thr37/46) in glioblastoma tissue (large image tumor border, small inlay image tumor center). (D) Phospho-stainings in the corresponding spheroid culture according to different cutting levels within the cell pellet. (E) Longitudinal section through a LNT-229 FFPE glioma cell pellet showing a strong gradient from superficial cell layers to the bottom of the pellet.

During the establishment of the panel of antibodies used for the detection of phospho-specific stainings, we observed a strong gradient from the tissue border to the center of the specimen, with almost no staining signals in the tissue center. To address this issue systematically we generated primary cultures from a GBM under spheroid conditions and generated a cell pellet, which we fixed in formalin (Fig 4A). Additionally, the major part of the tissue was processed for routine diagnostic analysis. The tumor as well as the cultured cells stained positive with our panel of phospho-mTOR-target-antibodies. Superficial tissue or superficial cell layers, that first came into contact with formalin showed the strongest signals for the tested antibodies, while tumor centers hardly stained positive (Fig 4B and 4C). Similar results were detected in the primary culture of the GBM with strongest staining signal in superficial cell layers and a gradual decrease of staining intensity in the lower parts of the cell pellet (Fig 4D). Using longitudinal sections through a LNT-229 glioma cell pellet we were even able to show an intensity reduction after 2 mm for all tested antibodies (Fig 4E), whereas total proteins of RPS6 and 4EBP1 did not present with a staining gradient neither in the investigated primary culture tumor spheroids nor in the longitudinal LNT-229 glioma cell pellet (S1 Fig)

### mTORC1-signaling is also detectable in the tumor stroma of malignant brain tumors

**Fig 5:**
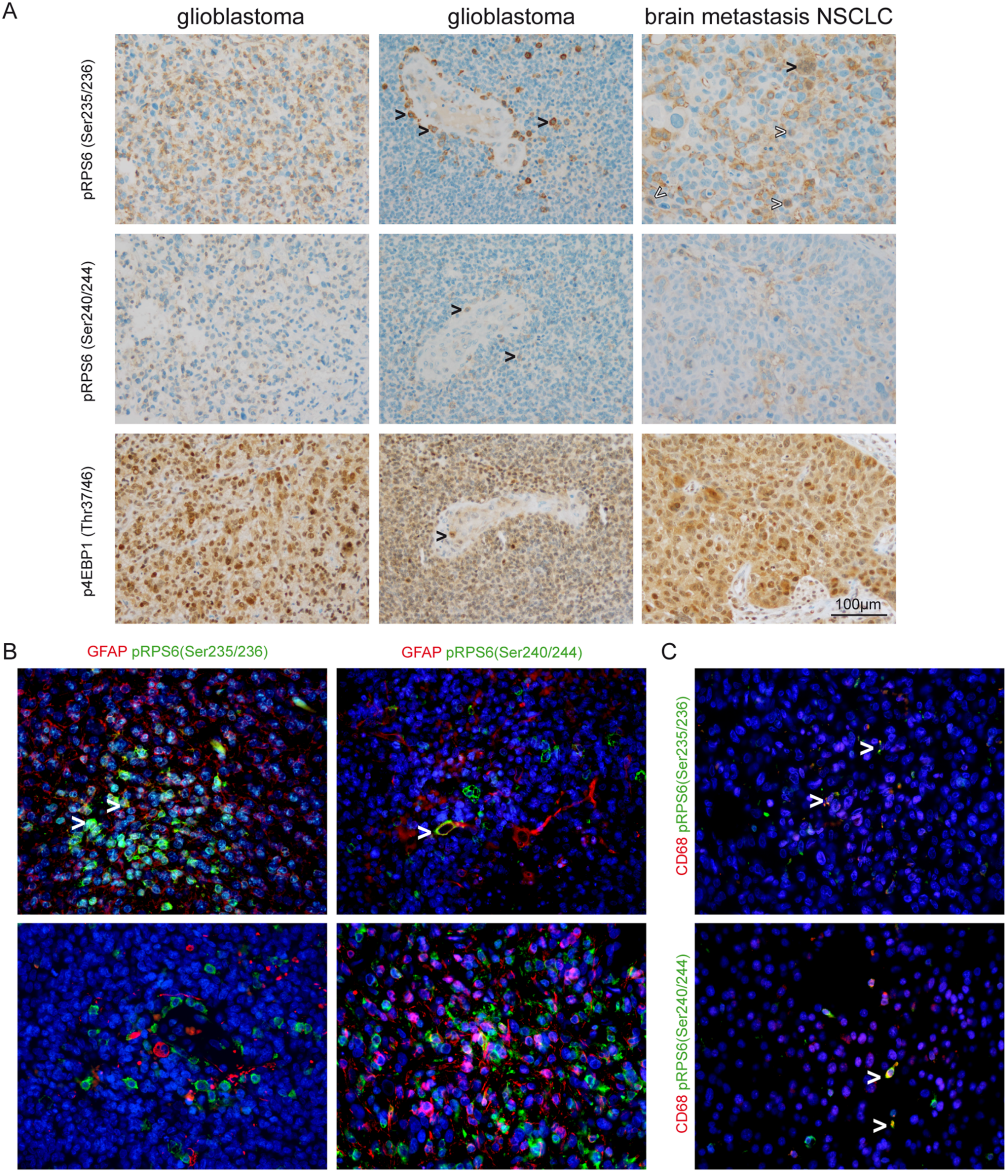
Detection of phospho-RPS6 (Ser235/236), phospho-RPS6 (Ser240/244) and phospho-4EBP1 (Thr37/46) in malignant brain tumors. (A) Although pleomorphic tumor cells showed staining for phospho-RPS6 (Ser235/236) and phospho-RPS6 (Ser240/244) we also observed perivascular cells with microglia/macrophage morphology that were strongly positive for both markers (black arrowheads in the first and second row of glioblastoma). We also detected strong expression of both phosphorylated antigens in brain metastases of NSCLC tumor cells (black arrowheads in the upper row, right column indicating multinucleated tumor cells, white arrowheads indicating mitotic figures). In contrast to the more heterogenous staining for phospho-RPS6 (Ser235/236) and phospho-RPS6 (Ser240/244), phospho-4EBP1 (Thr37/46) was strongly detected in the majority of tumor cells. Vessel-associated cells are also a source for mTORC1-signaling (black arrowhead indicating a mitotic figure within the endothelial layer, lower middle image). (B) Immunofluorescent double staining of glioblastomas against phospho-RPS6 (Ser235/236) and (Ser240/244) showing colocalisation with GFAP in some tumor cells (white arrowheads) while numerous GFAP-positive cells did not show phospho-signals for RPS6. (C) Phospho-RPS6 (Ser235/236) and (Ser240/244) were detected in CD68-positive cells in glioblastomas (white arrowheads).

As we already investigated SEGAs, as an example for a low grade brain tumor, we next focused on mTORC1-signaling in malignant brain tumors. Exemplarily, we analyzed tumor specimens from malignant primary brain tumors such as GBMs and secondary brain tumors i.e. brain metastases. A heterogeneous expression pattern was observed throughout the different specimens. Besides strong signals in pleomorphic tumor cells of carcinomas and GBMs we also observed strong expression in medium-sized mononuclear cells in perivascular areas, in smaller, rod-like cells as well as in endothelial cells. Immunofluorescent staining revealed a subgroup of CD68-positive cells that showed specifically positive signals for phospho-sites of RPS6. These cells were identified as CD68-positive microglia/macrophages (Fig 5).

### mTORC1-signaling is induced in glioblastomas as compared to normal brain

**Fig 6:**
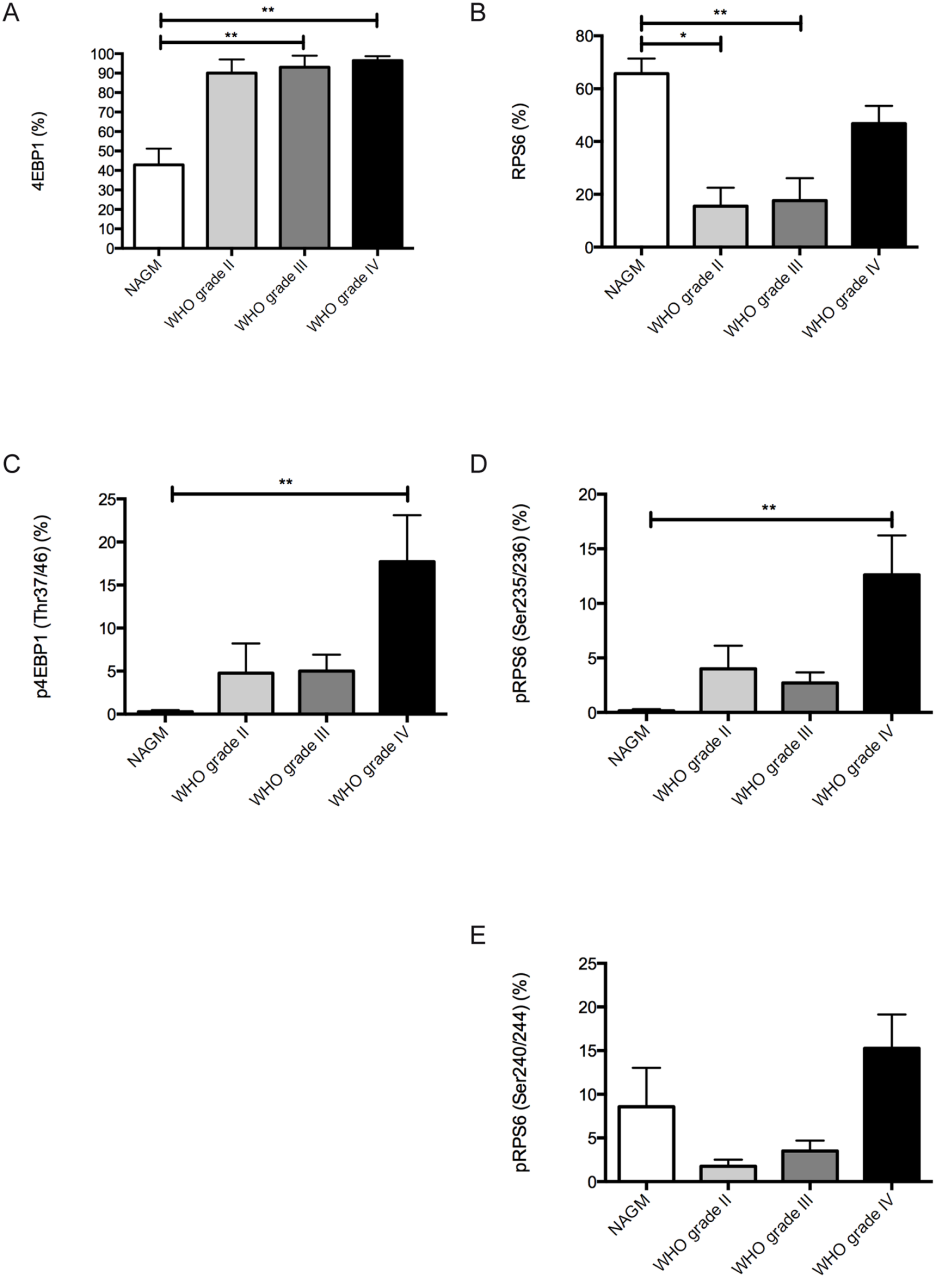
Quantification of mTORC1-signaling in glioma patients during tumor progression. All graphs show expression results of normal appearing grey matter (NAGM) tissue samples, WHO grade II gliomas, WHO grade III gliomas and glioblastomas (WHO grade IV). (A) Full 4EBP protein expression (NAGM vs. WHO grade III p = 0.001; NAGM vs. WHO grade IV p = 0.0003). (B) Full RPS6 protein expression (NAGM vs. WHO grade II p = 0.034; NAGM vs. WHO grade III p = 0.0072). (C) phospho-4EBP1 (Thr37/46) expression (NAGM vs. WHO grade IV p = 0.002). (D) phospho-RPS6 (Ser235/236) expression (NAGM vs. WHO grade IV p = 0.0018). (E) phospho-RPS6 (Ser240/244) expression. All other comparisons did not reveal statistically significant differences.

As a next step we were interested whether mTORC1-signaling is induced during tumor progression in glioma patients. Therefore we analyzed 15 patients who had either a low grade (grade II) or anaplastic (grade III) glioma as primary tumor, which then developed tumor progression to a GBM (grade IV) (Table 2). Interestingly, we only found a strong, significant induction of mTORC1-signaling in GBMs as compared to normal brain specimens (Fig 6). Although we also detected an induction of mTORC1-signaling in grade II and grade III gliomas, we did not find significant differences compared to normal brain or the GBM specimens. Surprisingly, 4EBP1 full protein counterparts were statistically increased in grade III gliomas and grade IV GBMs as compared to normal brain, whereas full RPS6 protein was significantly increased in the normal brain specimens as compared to grade II and grade III tumors (Fig 6A and 6B). Strongest signals for full RPS6 were detected in grey matter neurons.

## Discussion

The establishment of biomarkers reflecting individual oncogenic driver genes or proteins is of paramount importance in modern pathological diagnostics as well as oncological translational research and treatment. The mTOR-pathway is considered a promising candidate for targeted therapies because its relevance and activation has been demonstrated in a variety of cancers and specific inhibitors that have been tested in humans are readily available [26]. So far, potential biomarkers for mTORC1-signaling are RPS6 as well as 4EBP1. With regard to RPS6 and its phosphorylation at different sites, we included antibodies targeting both phosphorylations at the Ser235/236 and the Ser240/244 sites in our study. With regard to 4EBP1 we included the Thr37/46 phosphorylation site as this site phosphorylation interferes with the biological activity of 4EBP1 (i.e. binding of eIF4E) *in vivo* [19]. We found that phospho-RPS6 (Ser235/236), phospho-RPS6 (Ser240/244) and phospho-4EBP1 (Thr37/46) can be strongly detected in SEGAs as well as in malignant tumors of the central nervous system. Therefore these phosphoproteins represent potential candidates for predictive biomarkers or indicators of therapy response after mTORC1-targeted therapy. However, the use of phospho-specific antibodies for mTORC1 pathway components in routine diagnostics harbors the danger of several unique pitfalls. Here we provide a recommendation for a standardized approach to reduce misinterpretation of results.

### Suitability of phospho-RPS6 (Ser235/236), phospho-RPS6 (Ser240/244) and phospho-4EBP1 (Thr37/46) as mTORC1 biomarkers in FFPE tissue

We were able to show, that phospho-RPS6 (Ser235/236) and phospho-RPS6 (Ser240/244) staining intensities were reduced after rapamycin treatment and therefore both markers might serve as suitable biomarkers to detect an inhibition of mTORC1-signaling after rapamycin treatment in FFPE material. In contrast, we did not find a reduction of phospho-4EBP1 (Thr37/46) signals upon rapamycin treatment in FFPE material. Thus, phospho-4EBP1 (Thr37/46) is not suited to indicate activation of sensitive signaling or to monitor effective target inhibition for 1^st^ generation mTORC1 inhibitor therapy. In line with these findings, *in vitro* experiments using myotubes showed the absence of an effect on phosphorylation of 4EBP1 after rapamycin treatment [27]. Future therapy strategies might include newly designed second generation mTORC1-inhibitors such as torin2, which are ATP-competitive and work independent of FKBP12 [9,15]. These inhibitors exceed classical competitive mTORC1 inhibitors in their therapeutic potential most markedly indicated by the degree of 4EBP1 dephosphorylation. Accordingly, we were able to show an almost complete reduction in the amount of phospho-4EBP1 (Thr37/46) as well as reduced signal intensity for phospho-RPS6 (Ser235/236) and phospho-RPS6 (Ser240/244) in FFPE material after torin2 treatment. So far, there is no active clinical trial involving torin2, however *in vitro* and *in vivo* results using several cancer cell lines gave promising results [15,28]. Additionally, the drug has the great advantage of oral bioavailability [9].

### Time to formalin fixation as a limiting factor for the detection of phospho-RPS6 (Ser235/236), phospho-RPS6 (Ser240/244) and phospho-4EBP1 (Thr37/46)

Ideally, surgical specimens for neuropathological diagnostics are immediately fixed in formalin in the operation theatre. Sometimes, however, fixation of the removed tissue is delayed. In surgical pathology the fresh tissue specimen frequently undergoes a macroscopical analysis by the pathologist, which sometimes might take more than 30 minutes after removal of the specimen. We found that cell pellets of glioma cell lines showed a reduction of phospho-4EBP1 (Thr37/46) signal already after 30 minutes at room temperature without media. In contrast, the staining signal for both antibodies against phospho-RPS6 remained stable over 60 minutes in cell pellets and even within all tested intervals up to 230 minutes after removal of the surgical specimens. The strong reduction of phospho-4EBP1 staining signal is most likely due to early dephosphorylation events in the tissue, because total protein counterparts of RPS6 and 4EBP1 remained stable throughout the tested timeline. In general, this might be strongly dependent on (I) the investigated protein, (II) tissue temperature and (III) tissue type due to organ-specific fixative diffusion [29]. Therefore, we recommend an immediate fixation of tissue, and if this is not possible tissue should be stored on ice to reduce potential phosphatase activity. When storing the specimen on ice one should take care that the specimen does not freeze which might lead to morphological misinterpretations due to freezing artifacts.

### Tissue size is associated with a gradual reduction of phospho-signals for all tested antibodies

Fixation of tissue in formalin is a routine procedure successfully practiced since decades in (neuro-)pathology. However, although antibodies against routine antigens, e.g. against intermediate filaments GFAP or cytokeratins, are very robust in detecting their target in FFPE tissue, the time of penetration into the tissue of the fixative as well as the size of the tissue are crucial for successful detection of phospho-signals in FFPE material. We showed that larger pieces of tissue displayed a strong gradual reduction of staining intensity from the tissue border to the center and in addition we were able to show a strong reduction of staining intensity for all tested phospho-specific antibodies beyond 2 mm in a cell pellet generated from LNT-229 glioma cells, also here we observed a stable expression without a gradient for the RPS6 and 4EBP1 total proteins. These observations underline the importance for the use of defined tissue size to obtain homogenous staining results especially for phospho-RPS6 (Ser235/236), phospho-RPS6 (Ser240/244) and phospho-4EBP1 (Thr37/46). 4% formalin is known to penetrate 2–4 mm per 24 h into tissue [30], therefore a representative tissue sample should be cut not exceeding 4 mm in thickness before fixation with formalin. Best results showing almost no staining gradients derive from stereotactic brain biopsies which often do not exceed 2 mm in thickness (not shown). A decreasing phospho-staining gradient towards the tissue center indicates a potential underestimation of the actual degree of mTORC1 signaling and should therefore be taken into account whenever phospho-site specific stainings are evaluated. On the other hand a homogenous staining pattern across our mTORC1-antibody panel indicates adequate tissue processing.

### mTORC1 activity is not only detectable in the tumor cells but also in endothelial and immune cell subsets

Many targeted therapies are directed against oncogenic pathways, that are active in neoplastic cells, as for instance EGFR-, MET- or sonic hedgehog (shh)–signaling. This implies that the oncogenic signaling is exclusively active in neoplastic cells. Here, we showed that enhanced mTORC1-activity is also found in endothelial cells as well as in tumor-associated microglial/macrophages, especially in primary brain tumors such as GBMs. mTOR-activity has been described not only in the context of oncogenic signaling but has also been observed in reactive brain tissue especially in astrocytes and microglia after traumatic brain injuries [31] and stroke [32], while in case of glioma-activated microglia mTOR-inhibition polarized microglia to a M1-phenotype [33]. These aspects are of major importance when considering mTOR-activity as a biomarker for neoplastic cells. So far, it is not clear whether mTOR-targeted therapy additionally modulates the microenvironment through inhibiting angiogenesis or modifying polarization of microglial cells towards a tumor-inhibiting M1-phenotype in humans. The analysis of mTORC1-activity in patient tissue should be performed with caution since a high number of tumors also show strong expression of phospho-RPS6 (Ser235/236), phospho-RPS6 (Ser240/244) and phospho-4EBP1 (Thr37/46) on non-neoplastic cells in CNS tumors. The exact distribution of mTORC1-signaling in different primary as well as secondary brain tumors as well as the functional role of mTORC1-signaling in non-neoplastic cells strongly needs further investigation.

### mTORC1 activity as a potential marker for tumor progression in glioma patients

So far, mTOR-targeted therapies are not in clinical use for first line therapy in glioma patients, nevertheless in case of tumor relapse or progression mTOR-targeted therapy regimens might become important therapeutic options. Therefore we investigated glioma patients who had a grade II or grade III glioma as a primary tumor to test whether mTORC1 signaling was induced during tumor progression to GBM. Our results show an induction of mTORC1-signaling in GBM specimens as compared to normal brain specimens. During the progression course from grade II or grade III to grade IV (GBM) we found a strong trend for mTORC1-signaling induction, which supports the notion of mTORC1-activation as a driver for tumor progression. Nevertheless further studies are needed to examine the role of mTORC1-signaling in response to therapeutic interventions such as radio- and chemotherapy especially in GBM patients.

Target expression and relevance besides sufficient pharmacokinetics are a prerequisite for a successful targeted therapy. We found that the mTOR-pathway was activated in both human low grade brain tumors such as SEGAs, diffuse gliomas and malignant primary and secondary brain tumors. mTORC1 signaling is widely considered a relevant oncogenic pathway. Therefore mTOR-targeted therapies might be a useful strategy for brain tumor patients. Immunohistochemical stainings with antibodies against phospho-proteins phospho-RPS6 (Ser235/236), phospho-RPS6 (Ser240/244) and phospho-4EBP1 (Thr37/46) are suitable indicators for mTORC1-activity, however we recommend to follow our suggestions for tissue processing.

Finally only a panel of phospho-specific antibodies against phospho-RPS6 (Ser235/236), phospho-RPS6 (Ser240/244) and phospho-4EBP1 (Thr37/46) is adequate to gain reliable insights in mTORC1-activity in routine FFPE surgical specimens, especially when retrospectively or prospectively analyzing material of clinical trials.

## Supporting Information

S1 Fig Total protein counterparts of RPS6 and 4EBP1. Total RPS6 and 4EBP1 expression in: (A) archived SEGA specimens, (B) in vitro LNT-229 glioma cell fixation timeline, (C) in vivo GBM specimen fixation timeline, (D) primary glioma tumor spheres, (E) longitudinal section through a LNT-229 glioma cell pellet.
(TIF)
