# Supplementary figures and images for: Immunohistochemical Assessment of Phosphorylated mTORC1-Pathway Proteins in Human Brain Tumors
Source: PLoS One. 2015 May 19;10(5):e0127123. doi: 10.1371/journal.pone.0127123 (PMC4437987; doi:10.1371/journal.pone.0127123)

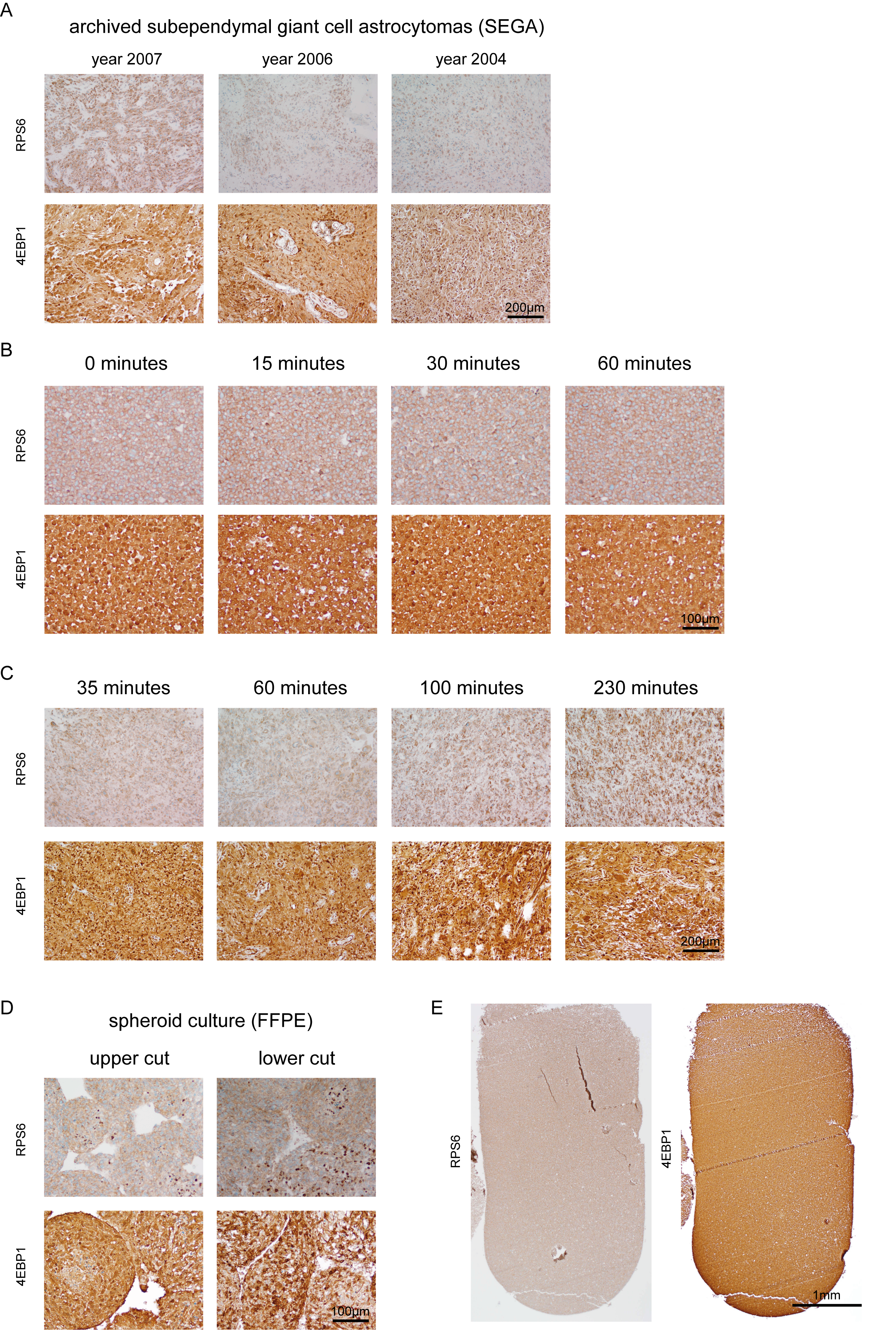

Supplement: S1 Fig — Total RPS6 and 4EBP1 expression in: (A) archived SEGA specimens, (B) in vitro LNT-229 glioma cell fixation timeline, (C) in vivo GBM specimen fixation timeline, (D) primary glioma tumor spheres, (E) longitudinal section through a LNT-229 glioma cell pellet. (TIF) [file pone.0127123.s001.tif]
